# Graphene/Silver Nanowires/Graphene Sandwich Composite for Stretchable Transparent Electrodes and Its Fracture Mechanism

**DOI:** 10.3390/mi12050512

**Published:** 2021-05-02

**Authors:** Chi-Hsien Huang, Hong-Cing Wu, Bo-Feng Chen, Yen-Cheng Li

**Affiliations:** 1 Department of Materials Engineering, Ming Chi University of Technology, New Taipei City 24301, Taiwan; a00820914@gmail.com (H.-C.W.); chses9551709@gmail.com (B.-F.C.); 2 Material and Chemical Research Laboratories, Industrial Technology Research Institute, Hsinchu 30011, Taiwan; yorkli@itri.org.tw

**Keywords:** graphene, sandwich structure, stretchable transparent electrode, artificial intelligence, wearable device

## Abstract

Polycrystalline graphene grown by chemical vapor deposition (CVD) is characterized by line defects and disruptions at the grain boundaries and nucleation sites. This adversely affects the stretchability and conductivity of graphene, which limits its applications in the field of flexible, stretchable, and transparent electrodes. We demonstrate a composite electrode comprised of a graphene/silver nanowires (AgNWs)/graphene sandwich structure on a polydimethylsiloxane substrate to overcome this limitation. The sandwich structure exhibits high transparency (>90%) and excellent conductivity improvement of the graphene layers. The use of AgNWs significantly suppresses the conductivity loss resulting from stretching. The mechanism of the suppression of the conductivity loss was investigated using scanning electron microscopy, atomic force microscopy, and lateral force microscopy. The results suggest that the high surface friction of the sandwich structure causes a sliding effect between the graphene layers would produce low crack or hole formation to maintain the conductivity. In addition to acting as conductive layers, the top and bottom graphene layers can also protect the AgNWs from oxidation, thereby enabling maintenance of the electrical performance of the electrodes over a prolonged period. We also confirmed the applicability of the sandwich structure electrode to the human body, such as on the wrist, finger, and elbow.

## 1. Introduction

Transparent conductive films (TCFs) are extensively used in electronic applications such as organic light-emitting diodes, liquid crystal displays, and touch screen panels [1,2,3,4]. Indium-doped tin oxide (ITO), which exhibits excellent physical properties, such as high transmittance (light penetration of over 80%) and very low sheet resistance (<30 Ω/□), is extensively employed in the electronics industry. Currently, with the advent of flexible and wearable electronic devices [5], TCFs are expected to exhibit flexibility, high conductivity, stability, and portability. The flexibility and stretchability of the TCFs would be critical due to its applicable applications, including human microchip implants, smart clothes, and smart portable devices. ITO films are brittle and crack easily upon bending or stretching. In addition, indium is a rare element that can only be found as a trace element in other minerals. As per the results, ITO does not meet the needs of flexible transparent devices. Therefore, researchers are investigating promising replacements for rigid ITO electrodes. Potential alternatives to the ITO substrate include conductive polymers, carbon nanotubes, graphene, silver nanowires (AgNWs), and other promising conductive transparent materials [6,7,8,9,10,11,12]. On the other hand, graphene exhibits many excellent properties, such as conductivity, mechanical strength, transmittance, and chemical stability [13,14,15]. These characteristics make graphene a great potential replacement for the commonly used ITO TCFs. Currently, the most common fabrication methods of graphene include mechanical exfoliation [16], epitaxial growth [17], chemical vapor deposition (CVD) [18], and chemical exfoliation for mass production of graphene oxide followed by reduction [19]. One of the best and easiest ways to obtain high-quality graphene films is by using CVD, which can be conveniently and effectively adopted in the roll-to-roll process for mass production [20]. This large-area membrane was then utilized in the field of transparent conductive electrodes. Graphene grown by CVD is generally polycrystalline, and the presence of grain boundaries gives rise to poor electrical properties in the film. CVD-grown graphene generally exhibits a higher sheet resistance than commercially available ITO films [21]. Therefore, it is imperative to improve the conductivity of graphene while maintaining its mechanical strength and optical transmittance. For instance, Khrapach et al. demonstrated that FeCl_3_ intercalated few-layer graphene exhibits excellent conductivity and optical properties (sheet resistance: ~9 Ω/□ and transmittance: ~84%) [22,23]. However, intercalation with FeCl_3_ of mechanically exfoliated few-layer graphene restricts its application as a large-area TCF.

Although various transparent graphene-based electrodes have been explored [23,24,25,26,27], the simultaneous achievement of high transparency, conductivity, and stretchability in the electrode remain challenging. The greatest challenge is the retention of electrical conductivity upon stretching [28,29]. Moreover, the CVD-synthesized graphene has a polycrystalline nature [30,31], and the presence of grain boundaries results in a significantly higher resistance in the graphene film than in the ITO film. Chen et al. previously employed AgNWs to minimize the high sheet resistance of the polycrystalline graphene film; however, the exposure of AgNWs to ambient moisture and oxygen resulted in their oxidation [32,33]. In this study, we demonstrate the unique composite structure as a transparent and stretchable electrode. AgNWs were inserted between two graphene layers to form a G/AgNWs/G sandwich structure. This unique structure effectively suppresses the oxidation of AgNWs and enables retention of the initial resistance in the electrode, even upon stretching. In addition, our sandwich-structured electrode exhibited low sheet resistance, light transmittance, and retained electrical conductivity upon stretching. The fracture mechanism is also discussed. This reflects the potential of our electrode for replacing ITO films in flexible optoelectronics technologies.

## 2. Materials and Methods

### 2.1. Fabrication Process of G/AgNWs/G Sandwich Structure

**Figure 1:**
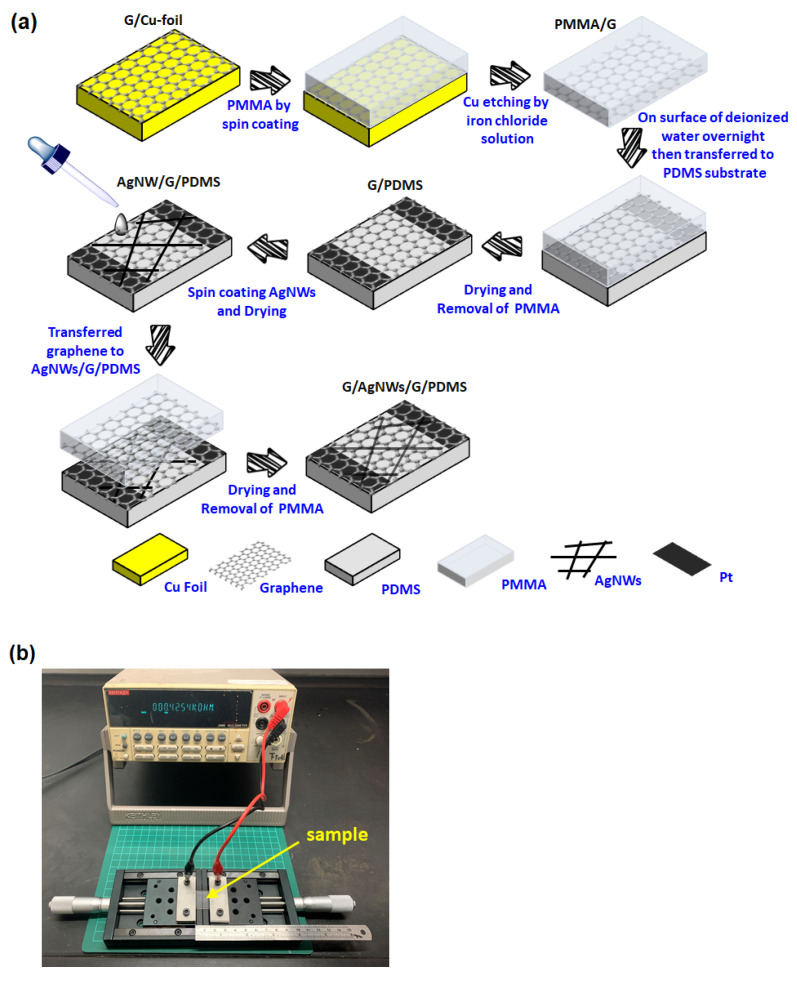
(**a**) Schematic of the fabrication process for the G/AgNW/G sandwich structure. (**b**) Photograph of the in-situ electrical resistance measurement during stretching.

Figure 1 schematically depicts the fabrication process of the G/AgNWs/G sandwich structure on the polydimethylsiloxane (PDMS) substrate. The process began with the synthesis of single-layer graphene on copper foil using CVD in a 3-inch-diameter tubular quartz furnace. The details were described elsewhere [34]. After synthesizing graphene, polymethyl methacrylate (PMMA) was spin-coated on the surface of the graphene/Cu-foil as a supporter. The dimensions of the graphene were 1.5 cm × 3 cm. Subsequently, the Cu foil was etched away using iron chloride. Then, graphene was rinsed in deionized water to remove the residual chemicals after the copper was completely etched to yield PMMA/graphene. Polydimethylsiloxane (PDMS) was cut into dimensions of 3 cm × 4.5 cm as a substrate for the stretching test. Then, the Pt electrodes were coated onto the PDMS substrate using a sputtering process. Subsequently, the PMMA/graphene was transferred onto the PDMS substrate. The sample was then dried at 80 °C on a hot plate. To remove PMMA from the surface of graphene, the sample was immersed in a solution of acetone and isopropyl alcohol (IPA). The samples were subsequently dried with nitrogen gas to form G/PDMS. The AgNW suspension (0.5 wt%; length: 30 μm; diameter: 115 nm) purchased from Aldrich was diluted with IPA (AgNW:IPA = 1:20 vol%) and then spin-coated on G/PDMS. The samples were then dried at 100 °C for 2 min to completely remove the solvent and obtain AgNWs/G/PDMS. Finally, another single layer of graphene was transferred onto the AgNWs/G/PDMS sample using the same method as the first transfer process to form a G/AgNWs/G sandwich structure on the PDMS substrate. Silicon dioxide/silicon (SiO2/Si) substrate were also used to investigate the quality of the CVD-grown graphene.

### 2.2. Characterization of G/AgNWs/G

Raman spectra were collected using a Horiba Raman system (iHR-550) with a wavelength of 532 nm. The Si peak at 520 cm^−1^ was used as a reference for wavenumber calibration prior to each measurement. The transmittance of the samples was measured using UV-Vis spectroscopy (V-650, JASCO Corp., Tokyo, Japan). Atomic force microscopy (AFM, Edge, Bruker Corp., Bill Rica, MA, U.S.) was employed to measure the lateral force and observe the surface morphologies, which were also characterized using a field emission scanning electron microscope (FESEM, JSM-6701F, JEOL Ltd., Tokyo, Japan). The samples were strained using a custom uniaxial strain rig equipped with current-voltage measurements (Keithley 2000, Tektronix, Beaverton, OR, U.S.) on the Pt electrodes to measure the in-situ electrical resistance during stretching. The electrical resistance variations were calculated as

(R − R_0_)/R_0_ (1)

where R is the electrical resistance under a certain strain, and R_0_ is the electrical resistance before strain.

## 3. Results and Discussion

### 3.1. Characterization of Sandwich Structure 

**Figure 2:**
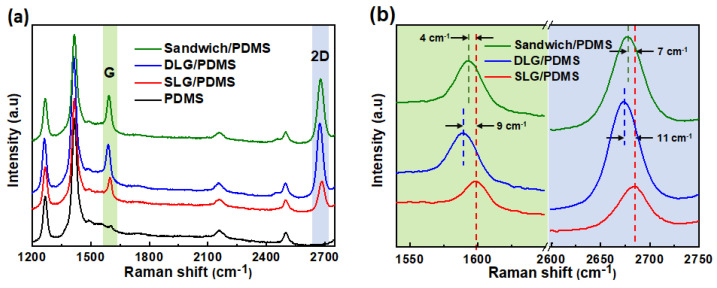
(**a**) Raman spectra of pristine PDMS, SLG/PDMS, DLG/PDMS and sandwich/PDMS samples; (**b**) enlargement of 2D peaks of SLG/PDMS, DLG/PDMS and sandwich/PDMS.

**Figure 3:**
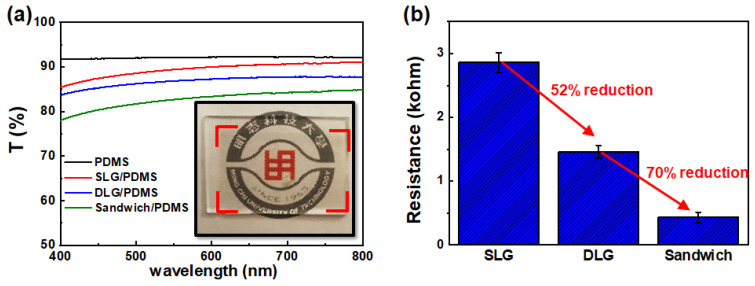
(**a**) UV-Vis spectrum of pristine PDMS, SLG/PDMS, DLG/PDMS and sandwich/PDMS. (**b**) Resistances of SLG, DLG, and sandwich.

**Figure 4:**
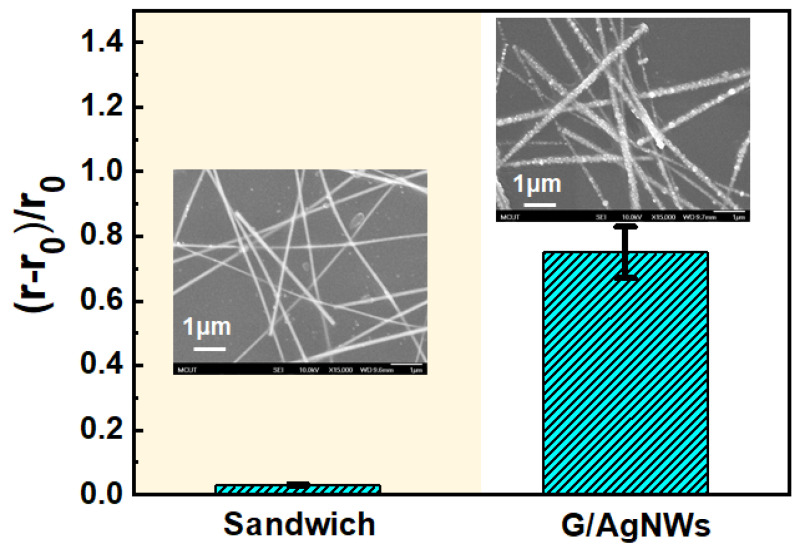
Resistance variation of the sandwich and AgNWs/G structures after exposing to the ambient for 18 h. Insets show the SEM images after exposure.

Before the preparation of the G/AgNWs/G sandwich structure, the quality of the CVD-grown single layer graphene (SLG) and double-layer graphene (DLG) were investigated using Raman and UV-Vis spectroscopies. A total of nine locations of a 3 cm × 3 cm graphene sheet were measured (see Figure S1). The Raman spectra of the nine points showed similar features (see Figure S2a); the G-band (ca. 1585 cm^−1^) and a 2D-band (ca. 2680 cm^−1^) were clearly observed representing the sp^2^-hybridized C-C bonds, while the D-band (ca. 1345 cm^−1^) representing atomic defects in graphene was almost invisible. The intensity ratio (I_2D_/I_G_) of the 2D- to G-bands was all above 2.0 (see Figure S2b). The average transmittances at 550 nm of the nine was 97.60% (see Figure S3a,b). Those results showed a high-quality and uniform SLG used in this study. The Raman spectrum of the stacked DLG featured an average I_2D_/I_G_ of 1.29 (see Figure S2c,d) lower than that of SLG indicating the formation of two-layer graphene due to the π–π interaction between them. The average transmittances at 550 nm of the DLG was 95.25% (see Figure S3c,d) which was almost consistent with the decrease of transmittance of 2.3% for SLG. Those results suggested high uniformity of SLG and DLG using the PMMA transfer method. To confirm the successful preparation of the G/AgNWs/G sandwich structure on the PDMS substrate, SLG/PDMS, DLG/PDMS, G/AgNWs/G sandwich structure (sandwich/PDMS), and PDMS substrate were analyzed by Raman spectroscopy, as shown in Figure 2a. The black curve denotes a typical Raman spectrum of pristine PDMS substrate, which correlates with that observed in previous studies [35,36]. The red curve denotes the representative Raman spectrum of SLG/PDMS. The appearance of the peaks at 1685 cm^−1^ (G band) and 2675 cm^−1^ (2D band) indicates the existence of graphene on the PDMS substrate [36]. The blue curve denotes the representative Raman spectrum of DLG/PDMS. We observed a blue shift of the characteristic peaks of the G and 2D bands after transferring one more layer onto SLG, as shown in Figure 2b, owing to the π–π interaction between the two layers of G [37,38]. The green curve denotes the representative spectrum of sandwich/PDMS. Evidently, the blue shift is smaller than that of DLG/PDMS. This result indicates that the interaction of AgNWs between the two layers of G weakens the π–π interaction between the layers. The top-view SEM image of the sandwich/PDMS (Figure S4) shows that the AgNWs were well distributed between the two layers of G. Given these results, we could confirm the successful preparation of the G/AgNWs/G sandwich structure. The optical and electrical properties of pristine PDMS, SLG/PDMS, DLG/PDMS, and sandwich/PDMS are shown in Figure 3. The optical transmittance of pristine PDMS was ~92.1%. After adding single layers of graphene onto PDMS, the optical transmittances of SLG/PDMS and DLG/PDMS were approximately 89.4% and 86.9%, respectively. SLG and DLG resulted in transmittance reductions of 2.6% and 5.5%, respectively, which are extremely similar to the theoretical transmittance of graphene. The transition of sandwich/PDMS at 550 nm decreased slightly to 83.2% when the AgNWs were inserted between the DLG structures. By excluding the light absorption of the PDMS substrate, the transmittance reduction resulting from the sandwich structure was only ~8.9%, indicating the potential for TCF application. The inset depicts a photograph of the sandwich/PDMS layer, which clearly reveals the underlying logo of our university. The resistances of the SLG, DLG, and sandwich between the two Pt electrodes were measured, as plotted in Figure 3b. The resistance of SLG was ~2.86 kΩ. After adding one more layer of graphene to form DLG, the resistance became 1.46 kΩ with a resistance reduction of 52% compared with SLG. The resistance of the sandwich structure significantly decreased to 0.43 kΩ with a resistance reduction of 70% and 85% compared with that of DLG and SLG, respectively. Notably, the conductivity of polycrystalline CVD-grown graphene is significantly influenced by the grain boundaries [39,40]. By adding AgNWs between the two layers of graphene to form a G/AgNWs/G sandwich structure, the AgNW bridges among the grain boundaries can significantly enhance the conductivity of this structure. Since AgNWs can form network pathways to bridge the polycrystalline CVD-grown graphene, they are unable to exist alone owing to easy oxidation, thereby resulting in significant conductivity reduction. Figure 4 shows the resistance variation denoted as r−r_0_/r_0_, where r is the electrical resistance after exposure to ambient conditions for 18 h, and r0 is the initial electrical resistance. It can be clearly seen that the resistance of AgNWs/G without graphene atop significantly increased by ~80% owing to the formation of silver oxide on the surface of the AgNWs, as shown in the SEM image in the inset of Figure 4. In contrast, the increased resistance of the sandwich structure, where the AgNWs were encapsulated between the two layers of graphene, was almost negligible; the surface of the AgNWs was very smooth, as shown in the SEM image in the inset of Figure 4. The results indicated that the graphene layer could protect the AgNWs from oxidation and provide good stability for practical applications.

### 3.2. Electromechanical Stretching Test

**Figure 5:**
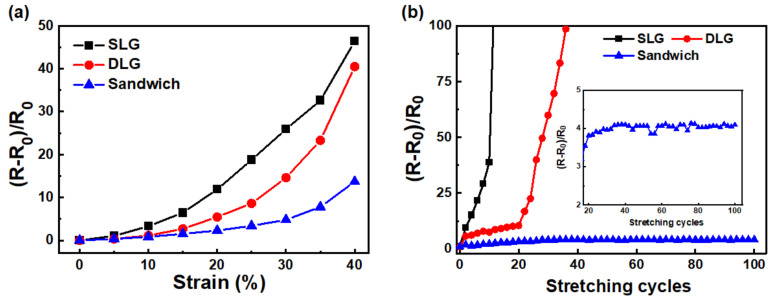
(**a**) Variation of electrical resistance with tensile strain. (**b**) Stretching stability under 20% strain for SLG, DLG and the sandwich structure.

**Figure 6:**
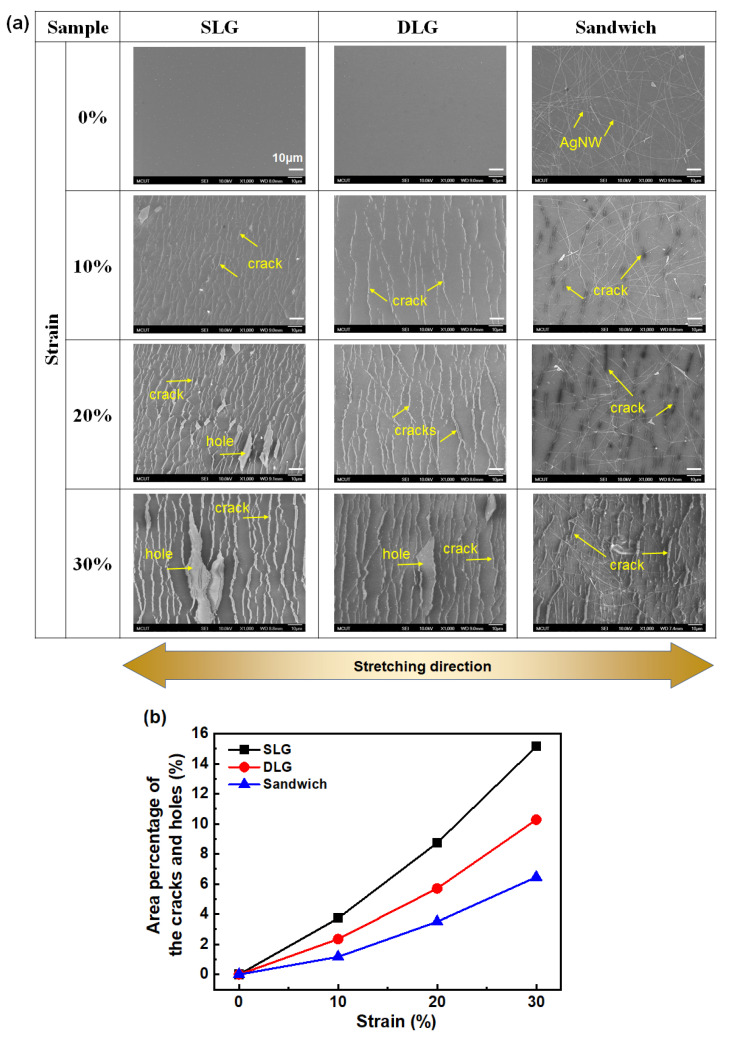
(**a**) SEM images and (**b**) area percentages of the SLG, DLG and sandwich structure under various tensile strain.

**Figure 7:**
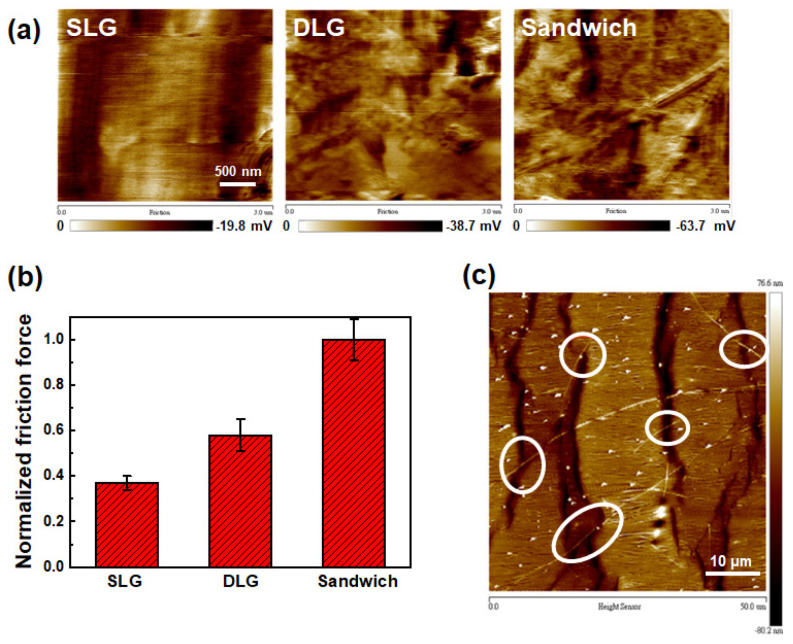
(**a**) LFM images and (**b**) normalized friction forces of the SLG, DLG, and sandwich structure; (**c**) AFM image of the sandwich structure under 30% strain.

The stretchable properties of the SLD, DLG, and sandwich structure were evaluated using the electrical resistance variation (R−R_0_)/R_0_ under various tensile strains, as shown in Figure 5a. The resistances of the three samples increased with increasing strain. Of the three samples, the resistance of SLG increased the fastest, while that of the sandwich structure increased slowly and linearly up to 30%. Stretching cycles were conducted at a constant strain of 20%, and the resistance variations were analyzed to demonstrate the stretching durability of the three samples. The resistance of SLG became unmeasurable after ~10 stretching cycles, whereas that of DLG behaved similarly after ~40 cycles. In contrast, the resistance variation of the sandwich structure gradually increased to ~4 after 20 stretching cycles. Thereafter, the conductivity of the sandwich structure stabilized up to 100 stretching cycles, thus demonstrating high stability. To investigate the mechanism of conductivity degradation and stability, we observed SEM images of the SLG, DLG, and sandwich structure and evaluated the area percentages of the cracks and holes under and holes under various tensile strains (Figure 6). A larger number of short cracks were created in SLG under 10% strain than in pristine SLG. The cracks became wider and connected to adjacent ones to form longer cracks and even holes under 20% strain. The cracks further became wider and longer, and the holes occupied a larger area under 30% strain. The cracks were perpendicular to the loading direction and quite similar to the cracking of polycrystalline CVD-grown graphene on copper foil reported by Na et al. [41]. These cracks were initialized at grain boundaries and developed as the applied strain increased. The fracture formation of DLG was similar to that of SLG. The crack density was slightly lower and the hole formed under a higher strain in DLG than in SLG, indicating that one or more graphene layer could inhibit crack or hole formation. The sandwich structure exhibited considerably fewer and shorter cracks than DLSG and no holes were formed. The area percentages of the cracks and holes for the SLG, DLG, and sandwich structures under various strains were analyzed and plotted in Figure 6b. The area percentage of the cracks and holes of the sandwich (6.46%) was 43% and 63% of those of SLG (15.14%) and DLG (10.28%),respectively, indicating that the presence of AgNWs could effectively suppress crack formation. To further explore the mechanism for the suppression of crack or hole formation, lateral force microscopy (LFM) was performed to investigate the adhesion forces of the samples. All LFM images were obtained from backward scans; bright and dark colors indicate relatively lower and higher values of friction force, respectively. Figure 7a depicts the LFM images of the SLG, DLG, and sandwich structures, and the normalized friction forces are plotted in Figure 7b. The normalized friction force of SLG is lower than that of DLG, which correlates with the results of previous studies [42], because the artificially stacked CVD graphene has a random misorientation among the layers, resulting in a relatively lower interaction between them. Therefore, the friction of DLG can increase owing to the puckering effect. The weaker interlayer interaction may cause a sliding effect between the layers during stretching. The sliding effect could release the overall tensile stress on DLG, resulting in fewer cracks than SLG. The sandwich structure exhibits an even higher value of normalized friction force, indicating a lower interaction due to the presence of AgNWs between the graphene layers.

The same phenomenon was also observed in the Raman spectra (Figure 2), where a redshift was detected. Therefore, a greater sliding effect could release more strain stress coming from the PDMS substrate, resulting in fewest cracks among the three samples. In addition to the suppression of the crack and hole formations, which causes low electrical conductivity loss during tensile strain, the bridge function of the AgNWs across the cracks was also confirmed, as shown in Figure 7c. AgNWs crossed the cracks when the sample was subjected to 30% strain. This result provides another reason for the retention of electrical conductivity during stretching.

### 3.3. Potential Applications for Wearable Devices

**Figure 8:**
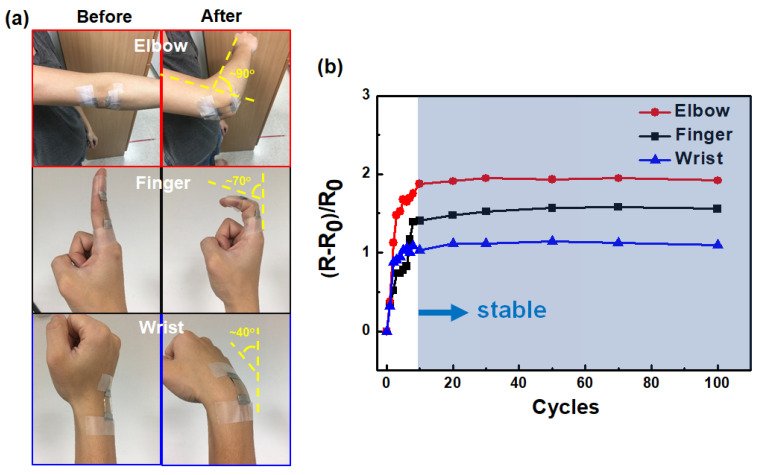
(**a**) Optical images of the sandwich structure placed on different parts of human body and (**b**) the corresponding variations of electrical resistance under stretching cycles.

To examine the practical use of wearable devices, a sandwich structure was placed on parts of the human body such as the elbow, finger, and wrist, as shown in Figure 8a. On these human joints, transparent electrodes are frequently subjected to stretching or bending. The sandwich structures were subjected to various cycling bending angles of approximately 90°, 70°, and 40° for the elbow, finger, and wrist, respectively, indicating various extents of stretching cycles. For all human joints, the sandwich structures stabilized after 10 cycles, as shown in Figure 8b. Notably, the value of the resistance variations depends on the part of the human joints, indicating that the sandwich structure is able to distinguish the extent of stretching in practical applications.

## 4. Conclusions

In summary, we developed a sandwich structured transparent electrode based on AgNWs intercalated between two graphene layers onto a PDMS substrate. This sandwich structure exhibited a high transmittance (>90%) and a significant resistance reduction of 85% in the CVD-grown SLG. In addition, the structure exhibited excellent long-term stability of the electrical properties under atmospheric conditions. The SEM and LFM results revealed that the high surface friction force induced a sliding effect owing to the presence of the AgNWs to release the strain stress, resulting in considerably less crack formation. The area of cracks and holes of the sandwich was only 43% of that of SLG. Therefore, the conductivity of the sandwich structure can be maintained during stretching. Finally, we demonstrated the potential applicability of the sandwich structure in wearable devices. The results revealed an inherent electrical stability, even after 100 stretching cycles on the human joints.

## Supplementary Materials

The following are available online at https://www.mdpi.com/article/10.3390/mi12050512/s1, Figure S1: Schematic of nine measuring locations in 3 cm × 3 cm SLG and DLG. Figure S2: (**a**) Raman spectra and (**b**) I2D/IG ratios of nine measuring points for SLG; (**c**) Raman spectra and (**d**) I2D/IG ratios of nine measuring points for DLG. Figure S3: (**a**) UV-Vis spectra and (**b**) transmittances of nine measuring points at 550 nm for SLG; (**c**) UV-Vis spectra and (**d**) transmittances of nine measuring points at 550 nm for DLG. Figure S4: Top-view SEM image of the sandwich/PDMS.
