# Supplementary material for: Graphene/Silver Nanowires/Graphene Sandwich Composite for Stretchable Transparent Electrodes and Its Fracture Mechanism
Source: Micromachines (Basel). 2021 May 2;12(5):512. doi: 10.3390/mi12050512 (PMC8147451; doi:10.3390/mi12050512)
Supplement: Supplementary file 1 [file micromachines-12-00512-s001.zip › micromachines-1169573-supplementary.pdf]

Supporting Information

Graphene/silver nanowires/graphene sandwich composites for stretchable transparent electrodes and its fracture mechanism

Chi-Hsien Huang [1,*], Hong-Cing Wu [1], Yen-Cheng Li [2]

[1] Department of Materials Engineering, Ming Chi University of Technology, New Taipei City 24301, Taiwan

[2] Material and Chemical Research Laboratories, Industrial Technology Research Institute, Hsinchu 310, Taiwan

* Corresponding author.

E-mail: chhuang@mail.mcut.edu.tw (C.-H. Huang)

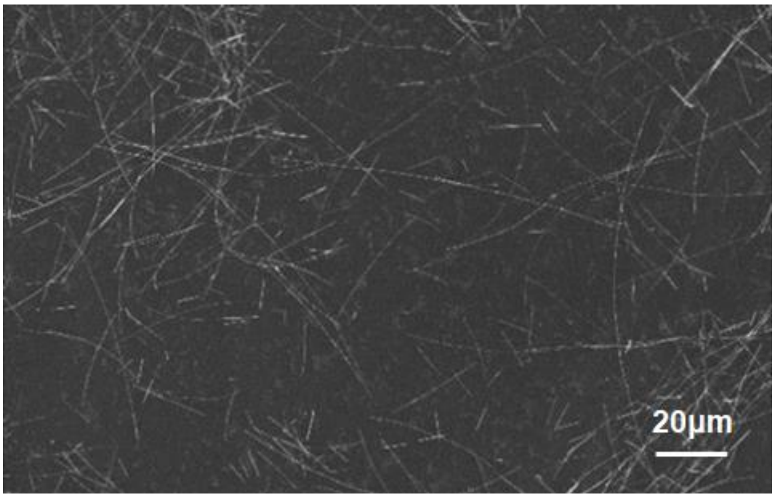

Figure S1 Top-view SEM image of the sandwich/PDMS.
